# Retraction: Downregulation of the CD151 protects the cardiac function by the crosstalk between the endothelial cells and cardiomyocytes via exosomes

**DOI:** 10.1371/journal.pone.0349178

**Published:** 2026-05-12

**Authors:** The PLOS One Editors

After this article [1] was published, concerns were raised regarding results presented in Figs 3-5 and S2.

Specifically:

- The following sets of panels appear to fully or partially overlap despite being used to represent different experimental conditions:
  - Fig 3f lipo and Fig 5b lipo
  - Fig 3f si-NC, Fig 5b si-NC, and Fig 5b con
  - Fig 3f si-CD151, Fig 5b si-CD151, and Fig 4d Ad-con
- The following panels appear to partially overlap:
  - Fig 3g con 0h and Fig 3g si-NC 0h
  - Fig 3g con 16h and Fig 3g lipo 16h
- In the Fig S2B TAC-GFP panel, there appears to be a vertical discontinuity between the peaks, and there appear to be irregularities in the bottom left of the image.

The corresponding author stated that Fig 3f in [1] is incorrect. Regarding the above concerns for Figs 3g, 4d, and 5b, the corresponding author stated that errors occurred in the preparation of these figures but did not state which of the published images are correct or incorrect. The corresponding author provided updated Figs 3f, 3g, 4d, and 5b, including updated associated quantified results, and original image data underlying these figures. Upon editorial review, PLOS identified additional concerns for the underlying data provided that call into question the reliability of the reported results in Figs 3-5.

Regarding Fig S2B, the corresponding author stated that the regions displayed in the published panel were selected from between peaks which had peak markers automatically generated by the analysis software, and that the peaks with these markers were removed, creating the appearance of a visual discontinuity in the background signal. They provided the original data underlying Fig S2B. Upon editorial review of these data, the *PLOS One* Editors noted additional concerns that call into question the reliability of Fig S2B in [1].

In light of the above unresolved concerns, the *PLOS One* Editors retract this article.

LJ, JL, ZY, JW, WK, and HZ agreed with the retraction. KZ responded but expressed neither agreement nor disagreement with the editorial decision. CZ either did not respond directly or could not be reached.
